# Adherence to Follow-Up Recommendations by Triathlon Competitors Receiving Event Medical Care

**DOI:** 10.1155/2017/1375181

**Published:** 2017-01-19

**Authors:** Jeremy D. Joslin, Jarem B. Lloyd, Nikoli Copeli, Derek R. Cooney

**Affiliations:** ^1^Department of Emergency Medicine, State University of New York Upstate Medical University, Syracuse, NY, USA; ^2^Department of Orthopedics and Sports Medicine, St. Luke's University Health Network, Bethlehem, PA, USA; ^3^Department of Emergency Medicine, New York Hospital Queens, Flushing, NY, USA; ^4^Department of Emergency Medicine, 550 East Genesee St., Syracuse, NY 13210, USA

## Abstract

*Introduction*. We sought to investigate triathlete adherence to recommendations for follow-up for participants who received event medical care.* Methods*. Participants of the 2011 Ironman Syracuse 70.3 (Syracuse, NY) who sought evaluation and care at the designated finish line medical tent were contacted by telephone approximately 3 months after the initial encounter to measure adherence with the recommendation to seek follow-up care after event.* Results*. Out of 750 race participants, 35 (4.6%) athletes received event medical care. Of these 35, twenty-eight (28/35; 80%) consented to participate in the study and 17 (61%) were available on telephone follow-up. Of these 17 athletes, 11 (11/17; 65%) of participants reported that they had not followed up with a medical professional since the race. Only 5 (5/17; 29%) confirmed that they had seen a medical provider in some fashion since the race; of these, only 2 (2/17; 12%) sought formal medical follow-up resulting from the recommendation whereas the remaining athletes merely saw their medical providers coincidentally or as part of routine care.* Conclusion*. Only 2 (2/17; 12%) of athletes who received event medical care obtained postrace follow-up within a one-month time period following the race. Event medical care providers must be aware of potential nonadherence to follow-up recommendations.

## 1. Introduction

Participation in triathlons has increased over the past two decades [1, 2]. With this rise in popularity, it is important to consider that triathlons have been associated with illness and injury including dehydration, cardiovascular complications, metabolic abnormalities, heat-related illness, and musculoskeletal injuries [2, 3]. USA Triathlon statistics have shown that the average age of triathlon participants is 38 years old, drawing the majority of participants from the 30–50-year-old age demographic: a group older than expected to participate in long duration, high intensity sports, and a group that may conceivably carry greater susceptibility to injury [1].

Factors such as experience, conditioning, environmental conditions, duration and distance of race events, and underlying athlete health can potentially contribute to inability to continue the race and result in the need for on-site medical evaluation [2, 3]. While some injuries occur during the course of the race, some participants may not become symptomatic until well after the athlete has finished the race and left the postrace area [4]. Over 85% of the triathletes seen in the medical area at the Kona Ironman Triathlon (Kailua-Kona, Hawaii) arrived at the main medical tent after finishing the race [4]. The concern for delayed presentation, or the existence of occult illness and injury, has also been expressed in a number of other published works in this area [5–7].

Similar to an emergency department, encounters at a race medical tent are often brief, and treatment guided towards resolution of acute symptoms. Whereas acute care provided in the emergency department setting may be more easily escalated to hospital admission or clinical referral, acute care in ultradistance medical tents is often limited by minimal supplies, limited infrastructure, with little-to-no established protocols besides hospital transfers by ambulance for true clinical emergencies.

Because medical encounters at race events are often limited and noncomprehensive, it is essential for patients to obtain follow-up care. Review of the current literature revealed no studies investigating how often patients who are evaluated in a race medical tent seek follow-up care when instructed to do so. Because of the importance of this follow-up care, the authors sought to investigate the adherence to recommendations that triathletes seek follow-up care after medical tent evaluation and treatment.

## 2. Methods

As part of a quality assurance review of athlete education on the importance of follow-up, participants of the 2011 Ironman 70.3 Syracuse (Syracuse, NY) who visited the designated finish line medical tent were evaluated by medical staff using a standardized encounter form which requested the athlete's follow-up contact information, as well as a checkbox to indicate their willingness to participate in our telephone follow-up at approximately three months after the event [IRB 294979-2].

The Ironman 70.3 Syracuse consists of three segments: a 1.2-mile swim, a 56-mile bike ride, and a 13.1-mile run. All triathlete participants treated in the medical tent were counseled to follow up with a medical provider for more definitive evaluation and to ensure resolution of symptoms. The participants were not advised of a specific time period to follow up. The medical encounter form used included a carbon copy tear-off sheet which was given to athletes upon medical tent discharge. All encounters were coded on the sheet with standard diagnoses (see the following list) at the time of evaluation. Athletes transferred off course or out of the medical tent by ambulance were excluded.

Standardized diagnoses used were

- mild to moderate heat-related illness;
- severe heat illness;
- dehydration or fatigue;
- gastric dysfunction;
- orthopedic or soft tissue injury;
- other.

Participants were contacted by telephone approximately 3 months after the initial encounter and asked if they did indeed follow up as instructed at their race medical tent evaluation. In the event that they did not follow up, they were asked to share the reason.

## 3. Results

Out of a total of 750 race participants, 35 (4.6%) athletes received event medical care at the medical tent. Of these 35, twenty-eight (28/35; 80%) consented to participate in the study and 17 (61%) were available to discuss their postevent care by telephone. Of these 17 athletes, 11 (11/17; 65%) of participants reported they had not followed up with a medical professional since the race. Only 5 (5/17; 29%) confirmed they had been seen by a medical provider since the race. Of these 5, only 2 (2/17; 12%) could be considered actual event medical follow-up, whereas the remaining athletes actually saw their medical providers coincidentally or as part of routine care. Athletes' own perception of symptom resolution was the most common reason among those who did not follow up. Results of the telephone interviews are summarized in Table 1.

## 4. Discussion

Endurance events demand sustained and elevated physiologic function under a variety of environmental conditions and therefore may incur a range of different injuries and physiologic insults. Common injury patterns seen at triathlon and other endurance events are well established in the literature [3, 8–11].

There are many examples in which an athlete's condition appears benign, yet requires diligent evaluation and should receive appropriate follow-up. These include, but are not limited to, subtle skeletal injuries that are only detected on radiographic imaging [12], hematuria that should be evaluated with serum biomarkers [10], and chest pain that should be risk stratified by electrocardiogram and serum marker assays due to related risk of cardiac events [13].

Although acute kidney injury (AKI) in this setting is typically self-limiting, an episode of severe AKI may warrant hospitalization and may not be caught until follow-up. McCullough found that 40% of marathon runners in a single event experienced only transient rises in creatinine level seen in AKI but suggests that chronic damage may accumulate in repeat participants [14]. Professional and amateur triathletes with repetitive AKI require the attention of follow-up care beyond the scope of the average event medical tent. Broad use of NSAIDs by athletes during endurance events may potentially increase the risk of AKI and exercise-associated hyponatremia, decreasing the glomerular filtration rate already lowered by exercise [15–17]. One urine dipstick method has been described to help risk stratify athletes with suspected renal injury at a medical tent [7].

Medical tents do not commonly have the capacity to perform radiographic examinations. One case series following stress fracture presentations among athletes found that diagnosis was delayed by an average of 3.5 months [18]. Our data reinforce the importance of follow-up care for orthopedic pain as one athlete reported a fracture found on radiographs at a follow-up visit (Athlete #7).

While there are clear differences between a race medical tent and an emergency department, the brief period of patient contact allowing a potential for complications after discharge is similar. In an emergency department study, leaving the patient to schedule their own follow-up was associated with poor follow-up adherence [19]. On the other hand, patients showed improved adherence among a comparison group where the follow-up appointment was prearranged [19]. Although it could be argued that not all athletes evaluated in a race medical tent require follow-up care, it may be difficult in the moment to stratify those who do and those who do not require that follow-up. In emergency medicine practice, it is common to recommend for these same reasons that all patients discharged from the emergency department be reevaluated in the near term.

Another factor found to improve adherence to recommended follow-up among self-pay and Medicaid patients was to provide a clinical appointment date before discharge [20]. In that subject pool where no appointment was provided, only 13% followed up with a medical provider. By comparison, in Roy Magnusson's study, where a prearranged clinical appointment was offered, approximately half of every comparison group followed up [19].

It may also be helpful to reflect on the “athlete attitude” which may include amplified feelings of health and wellness. Some athletes are likely to question a physician's or other provider's recommendation to obtain follow-up care, especially if the athlete has no prior relationship with the medical tent provider. Athletes may also carry feelings that they are “unbreakable” and too healthy to require medical care. Finally, it is not uncommon for athletes to simply not have a primary care provider if they have been generally healthy or are of a young age.

Considerations for improving follow-up rates include a primary follow-up care provider referral on the race registration form, documentation of participant primary care provider information on registration, or establishment of a designated follow-up provider for participants who visit the medical tent. Primary care sports medicine providers trained in both primary care and sports medicine offer an attractive option to triathletes requiring follow-up care [21]. The triathlete racer demographic possesses a more stable socioeconomic status [1], which presumably entails greater access to both insurance and follow-up medical care.

## 5. Limitations

This study represents a small sample size from a single event. The lack of comparable published literature limits the analysis to some degree as no other data set or expected rates of follow-up are known. Though a larger sample size may have enhanced the level of generalizability of the study, the results are consistent with common sense expectations and require further study. The unique practice environment typically involves care providers of various backgrounds, training, and licensure or certification. Because of this, it is not common practice to detail-oriented diagnoses. Typically general categories of field-expedient diagnoses are utilized, limiting potential subanalysis.

## 6. Conclusions

This study demonstrated that follow-up care recommendations were for the most part dismissed by athletes based on resolution of symptoms despite consistent medical staff advisement of all athletes who were seen in the medical tent to follow up with their healthcare professional. Athletes' perception of symptom resolution was the most common reason cited among those who did not follow up. Event medical care providers must be aware of potential nonadherence to recommendations and should consider potential strategies to improve follow-up compliance, especially in cases where the provider deems clinical follow-up to be important. Further studies are required to verify the causes of noncompliance with medical follow-up and the long-term outcome of subclinical triathlon or endurance sports injuries.

## Figures and Tables

**Table 1:** Follow-up characteristics of athletes.

Athlete	Complaint	Medical tent diagnosis	Medical tent intervention	Follow-up timeframe	Comments
1	Leg pain	(1) Orthopedic or soft tissue injury	(1) Comfort measures	3-4 weeks	Chronic iliotibial band pain

2	Paresthesias	(1) Other	(1) Comfort measures (2) Supplemental oxygen	None	Symptoms resolved so did not perceive need to follow up

3	Nausea & headache	(1) Dehydration or fatigue (2) Gastric dysfunction	(1) Comfort measures (2) Oral ondansetron (3) Oral hydration	None	Symptoms resolved so did not perceive need to follow up

4	Ankle pain	(1) Orthopedic or soft tissue injury	(1) Cold pack applied	2-3 months	Expected call from race staff to organize follow-up

5	Knee pain	(1) Orthopedic or soft tissue injury	(1) Cold pack applied	None	Symptoms resolved so did not perceive need to follow up

6	Nausea & dizziness	(1) Dehydration or fatigue	(1) Comfort measures (2) Oral ondansetron (3) Oral hydration	None	(1) Symptoms resolved so did not perceive need to follow up (2) Did not have PCP for follow-up

7	Fall with abrasion	(1) Orthopedic or soft tissue injury	(1) Topical antibiotic ointment (2) Cold pack applied (3) Wound dressing	2-3 weeks	Pain did not improve so sought follow-up care Diagnosed with fracture

8	Leg pain	(1) Orthopedic or soft tissue injury	(1) Cold pack applied	None	Symptoms resolved so did not perceive need to follow up

9	Fall with abrasion	(1) Orthopedic or soft tissue injury	(1) Topical antibiotic ointment (2) Cold pack applied (3) Wound dressing	None	Symptoms resolved so did not perceive need to follow up

10	Foot pain	(1) Orthopedic or soft tissue injury	(1) Comfort measures	2-3 months	Symptoms resolved so did not perceive need to follow up acutely

11	Knee pain	(1) Orthopedic or soft tissue injury	(1) Cold pack applied	None	Symptoms resolved so did not perceive need to follow up

12	Leg pain	(1) Orthopedic or soft tissue injury	(1) Comfort measures (2) Oral hydration	2-3 days	Symptoms persisted so followed up acutely

13	Blisters	(1) Orthopedic or soft tissue injury	(1) Topical antibiotic ointment (2) Wound dressing	None	Symptoms resolved so did not perceive need to follow up

14	Nausea & dizziness	(1) Dehydration or fatigue	(1) Comfort measures	None	Symptoms resolved so did not perceive need to follow up

15	Weakness & dizziness	(1) Dehydration or fatigue	(1) Comfort measures	None	Symptoms resolved so did not perceive need to follow up

16	Asthma exacerbation	(1) Other	(1) Inhaled beta agonist treatment	None	Symptoms resolved so did not perceive need to follow up

17	Itching	(1) Other	(1) Comfort measures	None	Symptoms resolved so did not perceive need to follow up
